# Simulated communication and teamwork scenario for nursing students: construction and validity

**DOI:** 10.1590/0034-7167-2024-0600

**Published:** 2025-12-08

**Authors:** Aline Teixeira Silva, Laura Andrian Leal, Maria Inês Lemos Coelho Ribeiro, Jeniffer Stephanie Marques Hilário, Monise Martins da Silva, Mateus Goulart Alves, Gabriela da Cunha Januário, Sílvia Helena Henriques

**Affiliations:** I Universidade do Estado de Minas Gerais. Passos, Minas Gerais, Brazil; II Universidade de São Paulo. Ribeirão Preto, São Paulo, Brazil

**Keywords:** Simulation Training, Interprofessional Education, Students Nursing, Communication, Patient Care Team., Entrenamiento Simulado, Educación Interprofesional, Estudiantes de Enfermería, Comunicación, Grupo de Atención al Paciente.

## Abstract

**Objectives:**

to construct, validate appearance and content, and test a simulated scenario for the development of communication and teamwork skills in nursing students.

**Methods:**

descriptive, methodological research, developed in three stages: scenario and checklist construction; scenario and checklist validity; and simulation validity. Data collection carried out between May 2020 and April 2021, after ethical approvals, with the participation of ten experts.

**Results:**

the scenario was called “Communication and teamwork in hospital environments”. The checklist, composed of briefing, scenario in action, and debriefing, after Content Validity Index, ended with 28 items. Subsequently, the simulation was validated by monitoring the scenario’s cinematography and the validated checklist.

**Conclusion:**

the scenario fulfilled the phases required for its creation and assessment, and can be used in the development of communication and teamwork in nursing students.

## RESUMEN

**Objetivos:**

construir, validar apariencia y contenido, y probar un escenario simulado para el desarrollo de comunicación y trabajo en equipo en estudiantes de enfermería.

**Métodos:**

investigación descriptiva, metodológica, desarrollada en tres etapas: construcción de escenarios y lista de cotejo; validación de escenarios y lista; y validación de simulación. Recolección de datos realizada entre mayo de 2020 y abril de 2021, luego de aprobaciones éticas, con la participación de diez expertos.

**Resultados:**

el escenario se denominó “Comunicación y trabajo en equipo en el ámbito hospitalario”. La lista de verificación, compuesta por *briefing*, escenario en acción y *debriefing*, después del Índice de Validez de Contenido, finalizó con 28 ítems. Posteriormente se validó la simulación mediante el monitoreo de la cinematografía del escenario y la lista validada.

**Conclusiones:**

el escenario cumplió con las fases requeridas para su creación y evaluación y puede ser utilizado en el desarrollo de habilidades los estudiantes de enfermería.

## INTRODUCTION

The scientific and technological development of the last ten years in the health field has demanded professionals who are increasingly analytical and active, capable of resolving the demands that arise in healthcare services^(1)^. In the hospital, a complex environment, the aim is to provide safe, quality care to the community through an interprofessional team that works collaboratively and in an integrated manner^(2)^.

In this complex sector, nurses play an essential role in relation to interprofessional collaboration^(3)^. It is known that interprofessionality comes from collaborative work, not just group work. Thus, nurses, as the team’s coordinating professional, need to acquire interprofessional skills, such as the ability to communicate and collaborate as a team. In this context, communication is seen as a crucial element for building good interpersonal relationships between caregivers, users and families. Teamwork, on the other hand, allows the integration of different professions and specialties^(4,5)^.

Therefore, scholars argue that training professionals with a focus on interprofessionality is advantageous for the acquisition of skills that contribute to collaborative practice^(6)^. It is also worth noting that interprofessional education, within universities, aims to work on professional recognition and mutual respect, ethics, clarity of roles, leadership, conflict management, communication, collaboration between the team, and comprehensive patient care^(7)^, in order to assist students and professionals in healthcare services.

Therefore, in order to ensure that future nurses improve their communication and teamwork skills during clinical practice, a teaching space must be created for this purpose^(8)^. In this situation, it is essential that training centers integrate active methods, such as simulation, with the aim of preparing students for practical experience and, thus, qualifying them for the job market.

In this line of thinking, simulation aims to put into practice a previously elaborated situation, through methodological processes, in a controlled and immersive environment, with the purpose of expanding participants’ experiences and providing the impression of a real context, in a specific environment^(9)^, thus contributing to the improvement of skills, knowledge and attitudes in students^(10)^. Clinical scenarios can be a valuable resource for guiding professors towards effective teaching and learning activities, and scenario validity is essential for creating situations that increasingly correspond to students’ practical reality^(11)^.

During the scenario validity process by experts in the field, all stages of scenario construction and preparation are assessed to ensure integrity, reproducibility and compliance with recommended clinical simulation standards. This assessment can be performed in the presence of an evaluator or remotely^(12)^. Thus, it is believed that the creation and approval of clinical scenarios that improve nursing students’ interprofessional skills are essential, although they are still incipient in many higher education institutions in Brazil.

Although clinical simulation is widely recognized, many studies focus on the assessment of clinical outcomes and the acquisition of technical skills, neglecting fundamental aspects of interpersonal relationships. Therefore, the lack of consolidated evidence reinforces the need for new studies that explore structured and validated approaches for the use of clinical scenarios in teaching communication and teamwork in nursing training. Such investigations are essential to support more effective educational practices aligned with healthcare demands, in which collaboration and communication are essential skills for safe praxis and quality of care.

## OBJECTIVES

To construct, validate appearance and content, and test a simulated scenario for developing communication and teamwork skills in nursing students.

## METHODS

This study comes from a doctoral thesis (https://doi.org/10.11606/T.22.2023.tde-07122023-140656).

### Ethical aspects

The research was approved by the Research Ethics Committee of the proposing institution and the co-participating institution, where data collection was conducted. All those involved signed the Informed Consent Form in two copies.

### Study design, period and location

This is descriptive and methodological research, developed in three stages: stage I - simulated scenario and checklist construction; stage II - scenario and checklist validity; and stage III - simulation validity. The article was written according to the Revised Standards for Quality Improvement Reporting Excellence 2.0^(13)^.

This study was carried out from May 2020 to April 2021 at a public higher education institution (HEI) in Minas Gerais. The simulated scenario was developed for students in their final year of undergraduate nursing courses^(14)^.

As a methodological framework, the Jeffries design model^(15)^ was chosen, which works on student support, simulation objectives, problem-solving, fidelity and debriefing. Furthermore, it focused on the International Nursing Association for Clinical Simulation and Learning^(16)^ guidelines, based on interprofessional education, respecting the briefing, scenario in action and debriefing phases.

### Population or sample; inclusion and exclusion criteria

Ten experts/evaluators participated in the study: five in the scenario and checklist validity stage and another five in the simulation validity stage. It is worth noting that the evaluators who participated in the scenario and checklist validity are different from those who participated in simulation validity. This is due to the fact that the former has more expertise in instrument validity and the others in simulated activities and interprofessionality. Evaluators were selected by convenience sampling. To define the evaluators, Fehring’s adaptation^(17)^ was used, focusing on academic training and experience in simulated teaching or interprofessionality. Experts were selected by analyzing their Lattes CV. The following scoring system was used to participate in the study: doctoral degree in nursing: 4 points; master’s degree in nursing: 3 points; dissertation in the area: 2 points; articles published in the area: 1 point; care practice or teaching in the area: 2 points; and specialization: 2 points. The required score was 7 points. An average of eight evaluators were invited to each validity stage via email; of these, ten agreed to participate, with five in each phase.

### Study protocol

In stage I, simulation scenario was constructed with details of each item. The scenario took into account the physical structure available in the institution’s laboratory, with the creation of a ward with basic materials and equipment, aiming to facilitate its reproduction in different realities without affecting the simulation’s complexity and fidelity. Subsequently, the checklist was constructed based on a study^(18)^, which presented four domains: briefing; scenario in action; debriefing; and overall assessment.

In stage II, the checklist and scenario were sent by email to five experts, requesting analysis and response within two weeks. There was no need for more than one round of validity. However, for experts who suggested several modifications, the scenario was forwarded, together with the corrected checklist, for a new assessment, establishing consensus of agreement.

After this process, preceding stage III, simulation was performed and filmed simultaneously in the skills laboratory of the HEI in question, with the authorization of all participants. Simulation involved the collaboration of six actors/directors, all healthcare workers, in order to facilitate the reproduction of the clinical practice scenario. Four of these professionals received prior training from the facilitator/researcher to perform their roles during the scenario: one as a simulated patient, another as a daughter/companion, one as a night shift nurse, and another as a physician. Two nurses represented students. It is important to emphasize that these two professionals did not receive any information or training about the simulation. They were instructed when they arrived at the laboratory that they would participate in a nursing simulation: one as a nurse and the other as a nursing technician. The participation of real students was prevented due to the pandemic period.

During the simulation, the researcher/facilitator, in possession of the scenario and the checklist, explained each stage of the scenario to participants/enactors: described the execution time, the scenario clinical case and objectives; presented the environment, materials and equipment available (briefing); and accompanied and directed participants during debriefing.

Finally, in stage III, the validated checklist and the footage were sent by email to the other five experts/evaluators for the simulation validity.

### Analysis of results and statistics

To validate the scenario and checklist, three criteria were analyzed: appearance; clarity; and relevance. Appearance is defined by the format of each part of the instrument. Clarity refers to the presentation of an easy and clear text to the reader. Relevance considers whether the text is relevant and/or appropriate^(19)^. Each element of the instrument could be scored from 1 to 5 points by experts according to a Likert scale, where 1 represents the lowest score and 5 the highest. A score above 4 met the research objectives^(19)^.

Subsequently, the Content Validity Index (CVI) was calculated. The CVI examines the degree of agreement of experts in relation to the simulated scenario activities and demands, considering a minimum of 0.80 as acceptable^(20)^. To analyze and align experts’ agreement, the AC1^(21)^ statistic was used, with a significance level of 5% (alpha = 0.05).

To validate the simulation, examiners, when watching the footage, marked on the checklist whether the participating professionals’ actions were “positive”, “negative” or “partially”. Experts were also able to make their suggestions on the instrument, if they considered it pertinent.

## RESULTS

To achieve the study objective, according to the methodological framework, the final scenario validated by experts is presented in Chart 1 below, describing the following topics: target audience; objectives; estimated time; briefing; scenario development; assessment; students’ expected actions; evolution; debriefing; environment; participants; and materials and equipment.

**Chart 1:** Simulated scenario: communication and teamwork in hospital environments, Ribeirão Preto, São Paulo, Brazil, 2023

Scenario: communication and teamwork in hospital environments
**Target audience:** students enrolled in the final year of a nursing degree
**General objective:** Provide nursing care to a hospitalized patient in a medical clinic unit, improving communication and team collaboration in the face of a given situation. **Specific objectives:** Analyze the patient’s general conditions and main complaint; review the patient’s medical records; identify the patient’s clinical history and antecedents; employ “interprofessional communication” and “teamwork” skills to resolve the patient’s problem; communicate assertively with the patient, family member and team to confirm information about the event that occurred; report the fact to the physician and assess possible medical procedures; record the event in the patient’s medical records.
**Estimated time:** briefing: 5 minutes; scenario: 10 minutes; debriefing: 20 minutes
**Briefing:** The facilitator introduces himself, establishes a confidentiality agreement and invites two students to participate in the scenario. He explains the main objective of the scenario to everyone present and informs the maximum time for the simulation to be carried out, allowing participants to familiarize themselves with the environment and the materials available. **Facilitator presents the clinical case to all participants:** Patient M. A. A., 76 years old, on the first day of hospitalization in the medical clinic with a diagnosis of pneumonia. She has a history of hypertension. There is a medical prescription and nursing progress in the patient’s medical record. The patient is conscious, with periods of confusion, requiring help to walk. She does not remember the names of the medications she takes continuously and does not remember if she has any allergies to any medications. On the unit’s daily schedule, there is a nursing technician (student) responsible for direct care to patient M. A. A., and a nurse (student) responsible for the unit. In the prescription room, there is the physician responsible for the patient, who is assessing the patient’s medical records to conduct the visit. It is now 7:15 am, and the nurse and the nursing technician receive the night shift nurse (actor).
**Scenario development:** **Shift handover:** Good morning! In room 102, bed 01, is patient Maria Aparecida Alves (fictitious name), 76 years old, on the first day of hospitalization with pneumonia. She is conscious, but has periods of confusion, requiring help to walk. She was admitted today at 5:00 am. The patient is hypertensive, but does not remember the names of her continuous-use medications. The medical prescription is already available. The patient is taking Levofloxacin 500 mg intravenously every 12 hours. At 6:30 am, the patient had a fever of 37.9 °C and was medicated with 40 drops of oral dipyrone. Her daughter, who lives in another city, has just arrived with the patient’s continuous-use medications and pressed the doorbell. Could you please answer the doorbell and write down the patient’s continuous-use medications in the medical record? Thank you! Have a good shift!
**Assessment:** A - Patent airway; B - Breathing room air; SPO_2_: 96%; RR: 18 bpm^a^; C - BP: 130/80 mmHg and HR: 95 bpm; D - Confused, uncommunicative; E - Peripheral venous access on the back of the right hand receiving 0.9% saline solution at 21 drop/min., without inflammatory signs. Fever peak (T: 37.9 ºC). Complains of pruritus in the chest and upper limbs.
**Expected actions from students:** Receive the shift handover; introduce yourself to the patient and her daughter; wash your hands; assess the patient’s general condition and main complaint; identify the itching in the patient’s upper limbs; ask questions about the patient and her daughter’s condition; confirm the prescribed medications and other information in the patient’s medical records; communicate effectively with the team and family, providing support when necessary; call the physician to assess the patient; prepare the prescribed medication after medical assessment; and record the patient’s continuous use medications, their allergy condition, as well as the nursing activities performed.
**Evolution:** Patient assessed and medicated after identification of allergy to dipyrone. The condition was stabilized, with cessation of pruritus.
**Debriefing:** **Main questions:** How did you feel when participating in the simulation? What emotions/feelings did you have when taking part in the scenario? Did you experience the scenario? Was the environment realistic? How do you rate your performance? What positive aspects do you highlight in your performance? Do your colleagues have any positive aspects to present? What aspects should be improved in your performance? What can your colleagues add? If the student does not reveal an attitude that was achieved, review their performance. What interprofessional competence was acquired in this context? (for those who participate and for those who observe); Do you believe that engaging in this learning activity will help you in practice during the practical internship? What is your colleagues’ view of the activity performed? **Synthesis:** the discussion should cover the stages of communication and team collaboration, personal presentation, comprehensive patient care, medication safety, recognition of allergy history, and decision-making. It is essential to highlight the importance of data collection at patient admission and interprofessional skills.
**Environment:** medical clinic sector.
**Participants:** a student in the role of a nurse; a student in the role of a nursing technician; an actor in the role of a night nurse; an actor in the role of a companion; an actor in the role of a simulated patient with moulage; an actor in the role of a physician.
**Materials and equipment:** hospital bed with ward sheets; bed identification form; chair for companion; medical records: medical prescription, nursing progress and vital signs; waste bin; bedside table; stethoscope; sphygmomanometer; portable oximeter; patient identification bracelet; patient identification in bed; watch; material for intravenous puncture and serum therapy; procedure gloves; medication tray; thermometer; patient medications; and makeup for moulage of the simulated patient.

*SPO_2_ - peripheral oxygen saturation; RR - respiratory rate; bpm^a^ - breaths per minute; BP - blood pressure; mmHg - millimeters of mercury; HR - heart rate; bpm - beats per minute; drop/min - drops per minute; T - temperature.*

The constructed checklist presented four domains (domain 1: briefing (7 items); domain 2: scenario in action (16 items); domain 3: debriefing (7 items); domain 4: overall assessment (4 items)), totaling 34 items.

The briefing domain consists of guidelines for participants to perform in the scenario, such as the number of participants, time, objectives, and environment presentation, materials, and equipment. In the scenario in action domain, the simulation itself is developed. In the debriefing domain, the facilitator encourages participants to conduct guided reflection, offering feedback and discussing points for improvement, associating theory with practice. In overall assessment, a space was made available for suggestions to experts.

The CVI was calculated based on experts’ assessment. The appearance, clarity and relevance criteria of each item of the instrument were assessed.

Concerning the briefing domain, the seven items showed an CVI higher than 80% (0.8), the minimum value to define the items as valid. Thus, all items were maintained. In the scenario in action domain, of the 16 items analyzed, two showed an CVI of 60% (0.6) in the appearance, clarity and relevance criteria, while the others showed an CVI of 80% (0.8). Therefore, the need to reformulate these items was taken into consideration. Thus, to adapt the scenario, evaluators’ suggestions were considered, making it more transparent, understandable and easy to replicate. Thus, the scenario in action domain ended up with 14 items. Finally, the debriefing domain obtained an CVI of 100% (1) for all items, without the need to change its structure, remaining unchanged.

The checklist validity was completed after the experts had checked, organized, corrected, and approved it. Chart 2 shows the final version of the checklist, which includes the briefing, scenario in action, and debriefing stages. It is important to note that participants’ actions, categorized as “positive,” “negative,” or “partially”, were maintained to facilitate replication with students. The fourth domain, referring to overall assessment (4 items), was removed from this version. The instrument ended up with 28 items. This version was used by the experts during the simulation validity.

**Chart 2:** Final checklist of the simulated scenario, Ribeirão Preto, São Paulo, Brazil, 2023

Domain Description	Item	Specification	Positive	Negative	Partially
Briefing	01	Facilitators introduce themselves to students.			
	02	A confidentiality agreement is established with students.			
	03	Facilitator invites two students to participate in the scenario.			
	04	The scenario’s overall objective is presented.			
	05	The scenario’s maximum execution time is presented.			
	06	The environment and material resources of the scenario are presented.			
	07	Facilitators present the clinical case to all participants. Afterwards, they leave the scene.			
Scenario in action	01	The two students, one playing the role of a nurse and the other as a nursing technician, receive the nursing shift from the night shift nurse (actor).			
	02	Students take patients’ medical records and go to patients’ bedside.			
	03	Students perform hand hygiene.			
	04	Students introduce themselves to patients and the daughter.			
	05	Students talk to patients and family about patients’ complaint.			
	06	Students assess patients’ general condition and identify patients’ allergy symptoms.			
	07	Students discuss patients’ condition.			
	08	Students confirm patients’ prescribed medications and other information in the medical record.			
	09	Students discuss patients’ allergy history with patients and family.			
	10	Students call the physician to the prescription room.			
	11	Students report the incident to a physician and check possible medical procedures.			
	12	After medical assessment, a nurse asks a nursing technician to prepare the prescribed medication after assessment.			
	13	Students work as a team to resolve the problem.			
	14	Students record patients’ continuous use of medications and their allergic condition as well as the nursing activities performed.			
Debriefing	01	Facilitator reiterates the simulation objective to the group.			
	02	Invites students to reflect on what was experienced in the simulated scenario.			
	03	Encourages students to highlight positive aspects of the scenario.			
	04	Encourages students to reflect on the possibility of behaving differently than they have been.			
	05	Discourages destructive criticism, if any.			
	06	Encourages students to discuss important aspects of communication and teamwork skills.			
	07	Encourages students to suggest other possibilities for communication and teamwork that were not presented in the scenario.			

Subsequently, simulation was validated. After analyzing the footage and the marked checklists, experts considered that the activities carried out in the briefing corresponded to the checklist, marking the actions as “positive” in the instrument.

In the context of scenario in action, which requires knowledge, skills and attitudes from participants, a divergence was identified in the assessments made by experts. In view of this, evaluators highlighted the importance of practicing hand hygiene and complying with Regulatory Standard 32 (no use of jewelry, hair tied up and well-trimmed nails). At the same time, one of the experts suggested applying makeup to simulate allergic reactions, allowing a more detailed analysis of patients’ skin condition and lesions. Additionally, two experts highlighted the need to identify patients accurately, using both the bracelet and the identification plate above the bed. They also reinforced the importance of ensuring safety in the medication administration process. Finally, it was recommended that the scenario only be concluded after students had recorded the information in patients’ medical record, including the progress observed and the verification of prescribed medications.

In the debriefing domain, which received 100% favorable responses, evaluators highlighted the need to reiterate the scenario objective, to highlight the importance of physical examination, which was not performed adequately, and to highlight the importance of communication and team collaboration as essential skills of nurses in the hospital context, illustrating with concrete actions and practical experiences in the health environment.

## DISCUSSION

In creating simulated clinical scenarios that are both valid and reliable, it is crucial to incorporate evidence-based methods, taking into account expert opinion to ensure that they are in accordance with recommended practices^(22)^, as demonstrated in this study. In addition, the development of these scenarios must be carefully planned and systematized, using instruments and tools that assist professors/facilitators during simulation^(23)^.

It is also important to ensure that each stage of the scenario is in agreement during the validity process. This offers the advantage of creating a more realistic clinical scenario aligned with the proposed topic^(19)^. In this context, the validity of the checklist carried out by experts gave more credibility to the scenario, bringing it even closer to reality.

During the scenario execution and filming for validity, three stages were implemented: briefing; scenario development; and debriefing. During the briefing, the first stage of simulation, as already described, it is important to present the environment, objectives, time and presentation of the case to participants. This information must be clear and objective, bringing students/professionals closer to the simulated reality^(24,25)^. During scenario in action, it is essential to offer support and guidance to the participants so that they can achieve the proposed objective of the simulation. In the meantime, it is essential to update the participants on the progress of the clinical case and the circumstances in which users/patients find themselves at that moment so that participants can understand their priorities^(26)^.

Simulation is recognized as a controlled environment that poses no risks to patients, closely resembling reality. This has contributed significantly to students recognizing their limitations and understanding the importance of acquiring solid theoretical knowledge essential to support practice^(27)^.

In debriefing, the last stage of the scenario, professors/facilitators must encourage students to share experiences and build new knowledge^(25,28)^. At this point, it is essential to identify emotions, remove doubts, highlight the skills and restrictions experienced by participants during the clinical situation and, simultaneously, encourage integration between theory and practice, generating greater interest in the subject discussed and reflection on the strategy applied in the clinical scenario^(29)^. Thus, during debriefing, the discussion addressed: communication and teamwork in relation to the handover; personal presentation; physical examination; assessment of patients’ complaint; analysis of medical records; patient safety in relation to hand hygiene; administration of medications; correct identification of patients; effective communication between patient/family and team; resolution of the problem presented; and nursing progress.

By feeling supported during simulation, students find it easier to explore and solve the issues presented in the scenario. Feedback from a professor is of great importance, as it allows them to reflect on their practices and future professional attitudes^(30)^. In this way, the experience lived by students during the simulation can lead to an in-depth understanding of the possible circumstances that may arise in nurses’ work, and this experience, carried out in a controlled environment, close to reality, corrects the errors and promotes greater security to work in the practical field^(31)^.

There are a number of advantages to adopting teaching strategies based on active methodologies, such as simulation, which do not necessarily require high technology to ensure successful learning^(32)^. Furthermore, traditional methods and simulations can be implemented together, helping the student to feel more confident and secure to act in practice^(33)^. Thus, the importance of disseminating validated clinical scenarios is highlighted so that HEIs can use them in their respective disciplines, further aiming at student learning^(12)^.

### Study limitations

A limitation is the specific scenario of the hospital sector, which may limit its application in other areas of healthcare. However, communication and teamwork are fundamental skills of nurses throughout their professional activities. Another limitation is due to the fact that the scenario was not tested/validated by experts in person in the skills/simulation laboratory with undergraduate students.

### Contributions to nursing

This study contributes to the development of scientific premises, since it allows undergraduate nursing courses to apply a validated scenario with an emphasis on communication and teamwork to students, allowing them to expand their knowledge through practical experience. Furthermore, this situation can offer a more comprehensive perspective of nurses’ work, which goes beyond care, leading these future professionals to a safe and collaborative practice in health institutions.

## CONCLUSIONS

Simulated scenarios stand out as an effective pedagogical resource that can be integrated into the educational curriculum, assisting students in their professional practice. Therefore, this study presented the development/creation and validity of a simulation on the interprofessional communication and teamwork skills of nurses in hospital environments. The validity, carried out by specialists, demonstrated the agreement of the assessments performed. The scenario and the checklist can be used free of charge in nursing education.

## Data Availability

The research data are available within the article.

## References

[1] Santos IH, Marum VL, Lanza LB. (2021). Innovative curriculum and labor market in the view of nursing graduates. Braz J Develop.

[2] Rosa CSR, Carvalho AGF, Barja PR. (2022). Soft skills: development of nursing skills nowadays. Rev Univap.

[3] Freitas CC, Mussatto F, Vieira JS, Bugança JB, Steffens VA, Baêta H (2022). Core competency domains in the collaborative practices of interprofessional health teams: an integrative literature review. Interface (Botucatu).

[4] Coifman AHM, Pedreira LC, Jesus APS, Batista REA. (2021). Interprofessional communication in an emergency care unit: a case study. Rev Esc Enferm USP.

[5] Peduzzi M, Agreli HLF, Silva JAM, Souza HS. (2020). Teamwork: revisiting the concept and its developments in inter-professional work. Trab Educ Saúde.

[6] Krielen P, Meeuwsen M, Tan ECTH, Schieving JH, Ruijs AJEM, Scherpbier ND. (2023). Interprofessional simulation of acute care for nursing and medical students: interprofessional competencies and transfer to the workplace. BMC Med Educ.

[7] Interprofessional Education Collaborative (IPEC) (2023). IPEC Core Competencies for Interprofessional Collaborative Practice: Version 3.

[8] Jackson MJ. (2024). Competency-Education and the Revised AACN Essentials: engaging practice leaders. Nurse Leader.

[9] Gaba DM. (2004). The future vision of simulation in health care. Qual Saf Health Care.

[10] Tamilselvan C, Chua SM, Chew HSJ, Devi MK. (2023). Experiences of simulation-based learning among undergraduate nursing students: a systematic review and meta-synthesis. Nurse Educ Today.

[11] Silva SCN, Alencar BR, Viduedo AFS, Ribeiro LM, Ponce de Leon CGRM, Schardosim JM (2021). Management of severe preeclampsia in the puerperium: development and scenario validation for clinical simulation. Rev Bras Enferm.

[12] Carvalho LR, Zem-Mascarenhas SH. (2020). Construction and validation of a sepsis simulation scenario: a methodological study. Rev Esc Enferm USP.

[13] Ogrinc G, Davies L, Goodman D, Batalden P, Davidoff F, Stevens D. (2015). SQUIRE 2.0 (Standards for QUality Improvement Reporting Excellence):revised Publication Guidelines from a Detailed Consensus Process. Perm J.

[14] Silva AT. (2023). Simulated experience for the development of communication skills and teamwork in undergraduate nursing students.

[15] Jeffries PR. (2012). Simulation in nursing education: from conceptualization to evaluation.

[16] Rossler K, Molloy M.A, Pastva AM, Brown M, Xavier N, INACSL Standards Committee (2021). Healthcare Simulation Standards of Best PracticeTM: simulation-enhanced interprofessional education. Clin Simul Nurs.

[17] Trojan RM, Sipraki R. (2015). Comparative studies from the application of the fourpoint likert scale: a methodological study of the Talis survey. Rev Ibero-Am Estud Educ.

[18] Ribeiro NM, Leal LA, Ferreira MVF, Chaves LDP, Ignácio DS, Henriques SH. (2023). Managerial decision-making of nurses in hospitals: creation and validation of a simulation scenario. Rev Latino-Am Enfermagem.

[19] Alexandre NMC, Coluci MZO. (2011). Content validity in the development and adaptation processes of measurement instruments. Ciên Saúde Coletiva.

[20] Polit DF, Beck CT. (2006). The Content Validity Index: are you know what’s being reported? critique and recommendations. Res Nurs Health.

[21] Gwet L. (2008). Computing Inter-Rater Reliability and its Variance in the Presence of High Agreement. Br J Math Stat Psychol.

[22] Au ML, Tong LK, Li YY, Ng WI, Wang SC. (2023). Impact of scenario validity and group size on learning outcomes in high-fidelity simulation: a systematics review and meta-analysis. Nurse Educ Today.

[23] Pedrollo LFS, Silva AC, Zanetti ACG, Vedana KGG. (2022). Creation and validation of a high-fidelity simulation scenario for suicide postvention. Rev Latino-Am Enfermagem.

[24] McDermott DS, Ludlow J, Horsley E, Meakim C, INACSL Standards Committee (2021). Healthcare Simulation Standards of Best PracticeTM Prebriefing: Preparation and Briefing. Clin Simul Nurs.

[25] Cowperthwait A. (2020). NLN/Jeffries simulation framework for simulated participant methodology. Clin Simul Nurs.

[26] Salifu DA, Christmals CD, Reitsma GM. (2022). Frameworks for the design, implementation, and evaluation of simulation-based nursing education: a scoping review. Nurs Health Sci.

[27] Al-Balawi I, Al-Qahtani JS, Al-Ghamdi SS, Al-Dhahir AM, Al-Nasser M, AlQ-ahtani AS (2022). Health Sciences Students’ Attitude, Perception, and Experience of Using Educational Simulation in Saudi Arabia: a cross-sectional study. Nurs Rep.

[28] Decker S, Alinier G, Crawford SB, Gordon RM, Wilson C, INACSL Standards Committee (2021). Healthcare Simulation Standards of Best PracticeTM: The Debriefing Process. Clin Simul Nurs.

[29] Johnson ME. (2021). Perceptions of higher education health science faculty on debriefing after simulation-based activities. J High Educ Theory Prac.

[30] White H, Hayes C, Axisa C, Power T. (2021). On the other side of simulation: evaluating faculty debriefing styles. Clin Simul Nurs.

[31] Coffey EK, McTier L, Phillips NNM. (2022). Final year undergraduate nursing students’ experience of high-fidelity simulation: results of a survey. Contemp Nurse.

[32] Santos ECN, Almeida RGS, Meska MHG, Mazzo A. (2021). Simulated patient versus high-fidelity simulator: satisfaction, self-confidence and knowledge among nursing students in Brazil. Cogitare Enferm.

[33] Tonapa SI, Mulyadi M, Ho KHM, Efendi F. (2023). Effectiveness of using high-fidelity simulation on learning outcomes in undergraduate nursing education: systematic review and meta-analysis. Eur Rev Med Pharmacol Sci.

