# Supplementary material for: Simulated communication and teamwork scenario for nursing students: construction and validity
Source: Rev Bras Enferm. 2025 Dec 8;78(Suppl 4):e20240600. doi: 10.1590/0034-7167-2024-0600 (PMC12700569; doi:10.1590/0034-7167-2024-0600)
Supplement: Translation

# Translation: Cenário simulado de comunicação e trabalho em equipe para estudantes de enfermagem: construção e validação

**DOI:** 10.1590/0034-7167-2024-0600pt

**Authors:** Aline Teixeira Silva, Laura Andrian Leal, Maria Inês Lemos Coelho Ribeiro, Jeniffer Stephanie Marques Hilário, Monise Martins da Silva, Mateus Goulart Alves, Gabriela da Cunha Januário, Sílvia Helena Henriques

**Affiliations:** I Universidade do Estado de Minas Gerais. Passos, Minas Gerais, Brasil; II Universidade de São Paulo. Ribeirão Preto, São Paulo, Brasil

**Keywords:** Treinamento por Simulação, Educação Interprofissional, Estudantes de Enfermagem, Comunicação, Equipe de Assistência ao Paciente.

## Abstract

**Objetivos:**

construir, validar aparência e conteúdo, e testar um cenário simulado para o desenvolvimento das competências de comunicação e trabalho em equipe em estudantes de enfermagem.

**Métodos:**

pesquisa descritiva, metodológico, desenvolvida em três estágios: construção do cenário e *checklist*; validação do cenário e *checklist*; e validação da simulação. Coleta de dados foi realizada entre maio de 2020 e abril de 2021, após aprovações éticas, com a participação de dez *experts*.

**Resultados:**

o cenário foi denominado “Comunicação e trabalho em equipe no ambiente hospitalar”. O *checklist*, composto por *briefing*, cenário em ação e *debriefing*, após Índice de Validade de Conteúdo, finalizou com 28 itens. Posteriormente, foi realizada a validação da simulação, por meio do acompanhamento da cinematografia do cenário e do *checklist* validado.

**Conclusões:**

o cenário cumpriu as fases exigidas para sua criação e avaliação, e poderá ser utilizado no desenvolvimento da comunicação e trabalho em equipe nos estudantes de enfermagem.

## INTRODUÇÃO

O desenvolvimento científico e tecnológico dos últimos dez anos no campo da saúde tem demandado profissionais que sejam cada vez mais analíticos e ativos, capacitados a resolver as exigências que surgem nos serviços de saúde^(1)^. No hospital, ambiente complexo, procura-se, através da equipe interprofissional, que atua de maneira colaborativa e integrada, oferecer um atendimento seguro e de qualidade à comunidade^(2)^.

Nesse setor complexo, o enfermeiro exerce papel essencial em relação à colaboração interprofissional^(3)^. Sabe-se que a interprofissionalidade advém do trabalho colaborativo, não somente grupal. Assim, o enfermeiro, profissional articulador da equipe, necessita da aquisição de competências interprofissionais, como a capacidade de se comunicar e de colaborar em equipe. A comunicação é vista neste contexto como um elemento crucial para a construção de um bom relacionamento interpessoal entre os cuidadores, usuários e família. Já o trabalho em equipe permite a integração das diversas profissões e especialidades^(4,5)^.

De tal modo, estudiosos defendem que a formação de profissionais com foco na interprofissionalidade é vantajosa para a aquisição de competências que contribuam para a prática colaborativa^(6)^. Ressalta-se ainda que a educação interprofissional, dentro das universidades, tem como objetivo trabalhar o reconhecimento e respeito mútuo profissional, a ética, a clareza de papéis, a liderança, o gerenciamento de conflitos, a comunicação, a colaboração entre a equipe, e o cuidado integral ao paciente^(7)^, a fim de auxiliar o estudante e os profissionais nos serviços de saúde.

Assim, visando garantir que os futuros enfermeiros aprimorem as capacidades de comunicabilidade e trabalho em equipe durante a prática clínica, deve ser criado um espaço de ensino para esta finalidade^(8)^. Nessa situação, é essencial que os centros formadores integrem métodos ativos, como a simulação, com o objetivo de preparar o discente para a experiência prática e, assim, habilitá-lo para o mercado de trabalho.

Nesse pensamento, a simulação visa colocar em prática uma situação previamente elaborada, por meio de processos metodológicos, em um ambiente controlado e imersivo, com a finalidade de expandir as vivências dos participantes e proporcionar a impressão de um contexto real, em um ambiente específico^(9)^, contribuindo, assim, para o aprimoramento das habilidades, conhecimentos e atitudes em estudantes^(10)^. Cenários clínicos podem ser um recurso valioso, visando orientar os docentes a atividades de ensino e aprendizagem eficazes, e a validação de cenários é fundamental para a criação de situações que correspondam cada vez mais à realidade prática dos estudantes^(11)^.

Durante o processo de validação do cenário por *experts* da área, todas as etapas da construção e preparação do cenário são avaliadas visando garantir a integridade, a reprodutibilidade e a conformidade com os padrões recomendados de simulação clínica. Esta avaliação pode ser realizada na presença do avaliador ou remotamente^(12)^. Desta forma, acredita-se que a criação e aprovação de cenários clínicos, que aprimorem as competências interprofissionais dos discentes de enfermagem sejam essenciais, embora ainda sejam insipientes em muitas instituições de ensino superior no Brasil.

Embora a simulação clínica seja amplamente reconhecida, muitos estudos se concentram na avaliação de resultados clínicos e na aquisição de habilidades técnicas, negligenciando aspectos fundamentais das relações interpessoais. Portanto, a ausência de evidências consolidadas reforça a necessidade de novos estudos que explorem abordagens estruturadas e validadas para o uso de cenários clínicos no ensino da comunicação e do trabalho em equipe na formação de enfermeiros. Tais investigações são essenciais para embasar práticas educacionais mais eficazes e alinhadas com as demandas do cuidado em saúde, em que a colaboração e a comunicação são competências essenciais para uma práxis segura e qualidade assistencial.

## OBJETIVOS

Construir, validar aparência e conteúdo, e testar um cenário simulado para o desenvolvimento das competências de comunicação e trabalho em equipe em estudantes de enfermagem.

## MÉTODOS

Este estudo é proveniente de tese de doutorado (https://doi.org/10.11606/T.22.2023.tde-07122023-140656).

### Aspectos éticos

A pesquisa foi aprovada pelo Comitê de Ética em Pesquisa da instituição proponente e da instituição coparticipante, onde a coleta de dados foi conduzida. Todos os envolvidos assinaram o Termo de Consentimento Livre e Esclarecido em duas vias.

### Desenho, período e localização do estudo

Investigação descritiva e metodológico, desenvolvida em três momentos: etapa I - construção do cenário simulado e *checklist*; etapa II - validação do cenário e *checklist*; e etapa III - validação da simulação. O artigo foi escrito conforme o *Revised Standards for Quality Improvement Reporting Excellence* 2.0^(13)^.

Este estudo foi realizado, no período de maio de 2020 a abril de 2021, em uma instituição de ensino superior (IES) pública de Minas Gerais. O cenário simulado foi desenvolvido para discentes do último ano de graduação em enfermagem^(14)^.

Como referencial metodológico, optou-se o modelo de *design* de Jeffries^(15)^, que trabalha o apoio ao estudante, os objetivos da simulação, a resolução de problemas, a fidelidade e o *debriefing*. Ainda, teve como foco as diretrizes da *International Nursing Association for Clinical Simulation and Learning* ^(16)^, com base na educação interprofissional, respeitando as fases de *briefing*, cenário em ação e *debriefing*.

### População ou amostra; critérios de inclusão e exclusão

Participaram da pesquisa dez *experts*/avaliadores: cinco na etapa de validação do cenário e *checklist* e outros cinco na etapa de validação da simulação. Destaca-se que os avaliadores que participaram da validação do cenário e *checklist* são diferentes dos que participaram da validação da simulação. Isso remete ao fato de os primeiros possuírem mais *expertise* em validação de instrumentos e os demais, em atividades simuladas e interprofissionalidade. Os avaliadores foram selecionados por amostra de conveniência. Para definir os avaliadores, empregou-se a adaptação de Fehring^(17)^, e teve com foco a formação acadêmica e a experiência no ensino simulado ou interprofissionalidade. A seleção dos *experts* foi realizada pela análise do Currículo *Lattes*. Utilizou-se a seguinte pontuação para participar do estudo: doutorado em enfermagem: 4 pontos; mestrado em enfermagem: 3 pontos; dissertação na área: 2 pontos; artigos publicados na área: 1 ponto; prática assistencial ou docência na área: 2 pontos; e especialização: 2 pontos. A pontuação exigida foi de 7 pontos. Foram convidados em média oito avaliadores para cada etapa de validação via e-mail; desses, dez se disponibilizaram em participar, sendo cinco em cada fase.

### Protocolo do estudo

Na etapa I, foi construído o cenário de simulação com detalhamento de cada item. O cenário levou em consideração a estrutura física disponível no laboratório da instituição, com a elaboração de uma enfermaria com materiais e equipamentos básicos, visando facilitar sua reprodução em diversas realidades sem afetar a complexidade e a fidelidade da simulação. Na sequência, foi construído o *checklist*, baseado em estudo^(18)^, que apresentava quatro domínios: *briefing*; cenário em ação; *debriefing*; e avaliação geral.

Na etapa II, foram encaminhados o *checklist* e o cenário por e-mail para cinco *experts*, solicitando análise e resposta avaliação em duas semanas. Não houve necessidade de mais de uma rodada de validação. No entanto, para os *experts* que sugeriram várias modificações, foi reencaminhado o cenário, juntamente com o *checklist* corrigido, para uma nova apreciação, estabelecendo o consenso de concordância.

Após esse processo antecedendo a etapa III, foram realizadas a execução e a filmagem simultânea da simulação no laboratório de habilidades da IES em questão, com autorização de todos os participantes. A simulação contou a colaboração de seis atores/encenadores, todos trabalhadores da saúde, a fim de facilitar a a reprodução do cenário da prática clínica. Quatro desses profissionais receberam treinamento prévio do facilitador/pesquisador para desempenhar suas funções durante o cenário: um como paciente simulado, outra como filha/acompanhante, uma como enfermeira do período noturno e outra como médica. Duas enfermeiras representaram os discentes. É importante ressaltar que essas duas profissionais não receberam qualquer informação ou treinamento sobre a simulação. Os mesmos foram orientados quando chegaram ao laboratório que iriam participar de uma simulação de enfermagem: um como enfermeiro e o outro como técnico de enfermagem. A participação de estudantes reais foi impedida devido ao período pandêmico.

Durante a execução da simulação, o pesquisador/facilitador, em posse do cenário e do *checklist*, explicou cada etapa do cenário aos participantes/encenadores: descreveu o tempo de execução, o caso clínico e os objetivos do cenário; realizou a apresentação do ambiente, materiais e equipamentos disponíveis (briefing); e acompanhou e direcionou os participantes durante o *debriefing*.

Por fim, na etapa III, o *checklist* validado e a filmagem foram encaminhados por e-mail aos outros cinco *experts*/avaliadores para validação da simulação.

### Análise dos resultados e estatística

Para a validação do cenário e *checklist*, foram analisados três critérios: aparência; clareza; e pertinência. A aparência é definida pelo formato de cada parte do instrumento. A clareza se refere à apresentação de um texto fácil e claro ao leitor. Já a pertinência considera se o texto é relevante e/ou apropriado^(19)^. Cada elemento do instrumento poderia ser pontuado de 1 a 5 pontos pelos *experts* conforme a escala Likert, em que 1 representa a pontuação mais baixa e 5, a mais alta. A pontuação acima de 4 atendia os objetivos da pesquisa^(19)^.

Posteriormente, realizou-se o cálculo do Índice de Validade de Conteúdo (IVC). O IVC examina o grau de consonância dos especialistas em relação às atividades e demandas do cenário simulado, considerando o mínimo 0,80 como aceitável^(20)^. Para analisar e alinhar a consonância dos especialistas, foi utilizada a estatística AC1^(21)^, sendo adotado o nível de significância de 5% (alfa = 0,05).

Para a validação da simulação, os examinadores, ao assistirem à filmagem, assinalavam no *checklist* a ocorrência das ações dos profissionais participantes em “positivo”, “negativo” ou “em partes”. Os *experts* também puderam realizar suas sugestões no instrumento, caso achassem pertinente.

## RESULTADOS

Para alcançar o objetivo do estudo, conforme o referencial metodológico, é apresentado no Quadro 1 abaixo o cenário final validado pelos especialistas, descrevendo os seguintes tópicos: público-alvo; objetivos; tempo estimado; *briefing*; desenvolvimento do cenário; avaliação; ações esperadas dos estudantes; evolução; *debriefing*; ambiente; participantes; e materiais e equipamentos.

**Quadro 1:** Cenário simulado: comunicação e trabalho em equipe no ambiente hospitalar, Ribeirão Preto, São Paulo, Brasil, 2023

Cenário: comunicação e trabalho em equipe no ambiente hospitalar
**Público-alvo:** estudantes matriculados no último ano de graduação em enfermagem
**Objetivo geral:** Fornecer assistência de enfermagem a um paciente hospitalizado em uma unidade de clínica médica, aprimorando a comunicação e a colaboração em equipe diante de uma situação apresentada. **Objetivos específicos:** Analisar as condições gerais e a queixa principal do paciente; revisar o prontuário do paciente; identificar a história clínica e os antecedentes do paciente; empregar as habilidades de “comunicação interprofissional” e “trabalho em equipe” para solucionar a intercorrência apresentada pelo paciente; dialogar de maneira assertiva com o paciente, familiar e equipe para confirmar informações sobre o evento ocorrido; informar o fato ao médico e avaliar possíveis condutas médicas; registar o evento no prontuário do paciente.
**Tempo estimado:** *briefing:* 5 minutos; cenário: 10 minutos; *debriefing:* 20 minutos
***Briefing:*** O facilitador se apresentam estabelece um acordo de confidencialidade e convida dois discentes a integrar o cenário. Expõe o objetivo principal do cenário a todos os presentes e informa o tempo máximo para a execução da simulação, permitindo que os participantes conheçam o ambiente e os materiais disponíveis. **Facilitador apresenta o caso clínico a todos os participantes:** Paciente M. A. A., de 76 anos, no 1° dia de internação na clínica médica com diagnóstico de pneumonia. Tem histórico de hipertensão arterial sistêmica. Há uma prescrição médica e evolução de enfermagem no prontuário do paciente. Paciente consciente, com períodos de confusão, demandando ajuda para deambular. A mesma não lembra dos nomes dos medicamentos de uso contínuo e não recorda se possui alergia de medicamentos. Na escala diária da unidade, há um técnico de enfermagem (estudante) responsável pelos cuidados diretos ao paciente M. A. A., e um enfermeiro (estudante) responsável pela unidade. Na sala de prescrição, encontra-se o médico responsável pelo paciente, o qual está avaliando os prontuários para realizar a visita. Agora são 07:15, e o enfermeiro e o técnico de enfermagem recebem o plantão da enfermeira do período noturno (ator).
**Desenvolvimento do cenário:** **Passagem de plantão:** Bom dia! No quarto 102, leito 01, encontra-se a paciente Maria Aparecida Alves (nome fictício), 76 anos, no 1° dia de internação com quadro de pneumonia. Está consciente, porém apresenta períodos de confusão, demandando ajuda para deambular. Ela foi internada hoje às 05:00. A paciente é hipertensa, porém não lembra o nome das medicações de uso contínuo. A prescrição médica já se encontra disponível. Paciente está fazendo uso de Levofloxacina 500 mg endovenosa de 12 em 12 horas. Às 06:30, a paciente apresentou pico febril de 37,9 °C e foi medicada com 40 gotas de dipirona via oral. A filha, que mora em outra cidade, chegou agora trazendo as medicações de uso contínuo da paciente e apertou a campainha. Por favor, vocês poderiam responder ao chamado desta campainha e anotar as medicações de uso contínuo da paciente no prontuário? Obrigada! Bom plantão!
**Avaliação:** A - Via aérea pérvia; B - Respirando ar ambiente; SPO_2_: 96%; FR: 18 rpm; C - PA: 130/80 mmhg e FC: 95 bpm; D - Confusa, pouco comunicativa; E - Acesso venoso periférico em dorso da mão direita recebendo soro fisiológico 0,9% a 21 gts/min., sem sinais flogísticos. Pico febril (T: 37,9 ºC). Queixa-se de prurido em tórax e membros superiores.
**Ações esperadas dos estudantes:** Receber a passagem de plantão; apresentar-se à paciente e a filha; higienizar as mãos; avaliar as condições gerais e a queixa principal da paciente; identificar o prurido nos membros superiores da paciente; realizar questionamentos quanto ao quadro apresentado junto à paciente e sua filha; confirmar as medicações prescritas e demais informações no prontuário da paciente; comunicar-se de forma efetiva com a equipe e familiares, fornecendo apoio quando necessário; chamar o médico para avaliar a paciente; preparar a medicação prescrita após avaliação médica; e realizar registro das medicações de uso contínuo do paciente, do seu quadro alérgico, bem como as atividades de enfermagem executadas.
**Evolução:** Paciente avaliada e medicada após identificação do quadro alérgico ao medicamento dipirona. O quadro foi estabilizado, com cessação do prurido.
***Debriefing:*** **Questões principais:** qual foi sua sensação ao participar da simulação? Qual emoção/sentimento ao fazer parte do cenário? Você vivenciou o cenário? O ambiente era verdadeiro? Como você considera seu desempenho? Quais são os aspectos positivos que você destaca em seu desempenho? Os outros colegas têm algum aspecto favorável para apresentar? Quais aspectos devem ser aprimorados em seu desempenho? O que os companheiros podem adicionar? Se o estudante não revelar uma atitude realizada, retome sobre a atuação do mesmo. Qual é a competência interprofissional adquirida nesse contexto? (para quem participa e para os que observam); Você acredita que engajar-se nessa atividade de aprendizagem irá auxilia-lo na prática durante o estágio prático? Qual é a visão dos colegas sobre a atividade realizada? **Síntese:** o debate deve abranger as etapas da comunicação e colaboração em equipe, apresentação pessoal, atenção integral ao paciente, segurança medicamentosa, reconhecimento do histórico alérgico, e tomada de decisão. É fundamental destacar a relevância da coleta de dados na admissão do paciente e das competências interprofissionais.
**Ambiente:** setor de clínica médica.
**Participantes:** um estudante no papel de enfermeiro; um estudante no papel de técnico em enfermagem; um ator no papel de enfermeiro do período noturno; um ator no papel de acompanhante; um ator no papel de paciente simulado com moulage; um ator no papel de médico.
**Materiais e equipamentos:** cama hospitalar com lençóis de enfermaria; ficha para identificação do leito; cadeira para acompanhante; prontuário: prescrição médica, evolução de enfermagem e sinais vitais; lixeira; mesa de cabeceira; estetoscópio; esfigmomanômetro; oxímetro portátil; pulseira de identificação do paciente; identificação do paciente no leito; relógio; material para punção endovenosa e soroterapia; luvas de procedimento; bandeja com medicação; termômetro; medicações do paciente; e maquiagem para *moulage* do paciente simulado.

*SPO_2_ - saturação periférica de oxigênio; FR - frequência respiratória; rpm - respirações por minuto; PA - pressão arterial; mmhg - milímetros de mercúrio; FC - frequência cardíaca; bpm - batimentos por minutos; gts/min - gotas por minuto; T - temperatura.*

O *checklist* construído apresentava quatro domínios (domínio 1: *briefing* (7 itens); domínio 2: cenário em ação (16 itens); domínio 3: *debriefing* (7 itens); domínio 4: avaliação geral (4 itens)), totalizando 34 itens.

O domínio *briefing* é composto por orientações aos participantes para a atuação no cenário, como o número de participantes, tempo, objetivos, e presentação do ambiente, materiais e equipamentos. Já no domínio cenário em ação, ocorre o desenvolvimento da simulação em si. No domínio *debriefing*, o facilitador estimula os participantes para uma reflexão guiada, oferecendo *feedback* e discutindo pontos de melhoria, associando teoria com a prática. Na avaliação geral, foi disponibilizado um espaço de sugestões para os *experts*.

Foi realizado o cálculo do IVC a partir da avaliação dos especialistas. Foram avaliados os critérios de aparência, clareza e pertinência de cada item do instrumento.

Em relação ao domínio *briefing*, os sete itens exibiram IVC superior a 80% (0,8), valor mínimo para definir os itens como válidos. Assim, todos os itens foram mantidos. No domínio cenário em ação, dos 16 itens analisados, dois apresentaram IVC de 60% (0,6) nos critérios de aparência, clareza e pertinência, enquanto os outros apresentaram IVC de 80% (0,8). Portanto, foi levada em consideração a necessidade de reformular esses itens. Assim, para adequar o cenário, as sugestões dos avaliadores foram consideradas, tornando-o mais transparente, compreensível e de fácil replicação. Dessa forma, o domínio cenário em ação finalizou com 14 itens. Finalmente, o domínio *debriefing* obteve um IVC de 100% (1) para todos os itens, sem a necessidade de alterar sua estrutura, mantendo-se inalterada.

A validação do *checklist* foi finalizada após a conferência, organização, correção e aprovação final dos *experts*. O Quadro 2 apresenta a versão final do *checklist*, que inclui as etapas de *briefing*, cenário em ação e *debriefing*. É importante destacar que as ações dos participantes, categorizadas como “positivo”, “negativo” ou “em partes”, foram mantidas para facilitar sua replicação com os estudantes. O quarto domínio, referente à avaliação geral (4 itens), foi removido desta versão. O instrumento finalizou com 28 itens. Essa versão foi utilizada pelos especialistas durante a validação da simulação.

**Quadro 2:** *Checklist* **final do cenário simulado, Ribeirão Preto, São Paulo, Brasil, 2023**

Descrição do domínio	Item	Especificação	Positivo	Negativo	Em partes
*Briefing*	01	Facilitador se apresenta aos estudantes.			
	02	É estabelecido acordo de confidencialidade com os estudantes.			
	03	Facilitador convida dois estudantes para participarem do cenário.			
	04	É apresentado o objetivo geral do cenário.			
	05	É apresentado o tempo máximo de execução do cenário.			
	06	São apresentados o ambiente e os recursos materiais do cenário.			
	07	Facilitador apresenta o caso clínico para todos os participantes. Após, sai de cena.			
Cenário em ação	01	Os dois estudantes, um no papel de enfermeiro e outro como técnico de enfermagem, recebem o plantão de enfermagem do enfermeiro do período noturno (ator).			
	02	Os estudantes pegam o prontuário do paciente e dirigem-se ao leito do paciente.			
	03	Os estudantes realizam a higienização das mãos.			
	04	Os estudantes apresentam-se à paciente e à filha.			
	05	Os estudantes conversam com a paciente e familiar sobre a queixa apresentada pela paciente.			
	06	Os estudantes avaliam as condições gerais do paciente e identificam o quadro alérgico da paciente.			
	07	Os estudantes discutem sobre o quadro da paciente.			
	08	Os estudantes confirmam no prontuário médico as medicações prescritas e demais informações do paciente.			
	09	Os estudantes conversam com a paciente e familiar sobre histórico de alergias da paciente.			
	10	Os estudantes chamam o médico na sala de prescrição.			
	11	Os estudantes comunicam o fato ocorrido ao médico e verificam possíveis condutas médicas.			
	12	Após avaliação médica, o enfermeiro solicita ao técnico de enfermagem que prepare a medicação prescrita após avaliação.			
	13	Os estudantes trabalham em equipe para resolutividade do problema.			
	14	Os estudantes realizam o registro das medicações de uso continuo do paciente e do seu quadro alérgico, bem como as atividades de enfermagem executadas.			
*Debriefing*	01	Facilitador reitera com o grupo o objetivo da simulação.			
	02	Convida os estudantes a refletir sobre o que foi vivenciado no cenário simulado.			
	03	Estimula os estudantes a levantar aspectos positivos realizados no cenário.			
	04	Estimula a reflexão dos estudantes sobre a possibilidade de condutas diferentes daquelas realizadas.			
	05	Desestimula críticas destrutivas, se houver.			
	06	Estimula os estudantes sobre aspectos importantes da competência comunicação e trabalho em equipe.			
	07	Estimula os estudantes a sugerir outras possibilidades de comunicação e trabalho em equipe que não foram apresentadas no cenário realizado.			

Posteriormente, foi realizada a validação da simulação. Após análise da filmagem e dos *cheklists* assinalados, os *experts* consideraram que as atividades realizadas no *briefing* corresponderam ao *checklist*, marcando as ações como “positivas” no instrumento.

No contexto do cenário em ação, que exige dos participantes conhecimento, habilidades e atitudes, foi identificada uma divergência nas avaliações feitas pelos especialistas. Diante disso, os avaliadores destacaram a importância de praticar a higienização das mãos e cumprir com a Norma Regulamentadora 32 (sem uso de adornos, cabelos presos e unhas bem cortadas). Paralelamente, um dos especialistas sugeriu a aplicação de maquiagens simulando reações alérgicas, possibilitando uma análise mais detalhada das condições da pele e das lesões apresentadas pela paciente. Além disso, dois especialistas ressaltaram a necessidade de identificar o paciente com precisão, utilizando tanto a pulseira quanto a placa de identificação acima do leito. Eles também reforçaram a relevância de garantir a segurança no processo de administração de medicamentos. Por fim, foi recomendado que o cenário só fosse encerrado após os estudantes registrarem as informações no prontuário da paciente, incluindo o progresso observado e a verificação das medicações prescritas.

No domínio *debriefing*, que obteve 100% de respostas favoráveis, os avaliadores destacaram a necessidade de reiterar o objetivo do cenário, de evidenciar a importância do exame físico, que não foi realizado adequadamente, e destacar a importância da comunicação e da colaboração em equipe como competências essenciais do enfermeiro no contexto hospitalar, ilustrando com ações concretas e experiências práticas do ambiente em saúde.

## DISCUSSÃO

Na criação de cenários clínicos simulados que sejam tanto válidos quanto confiáveis, é crucial incorporar métodos baseados em evidências, levando em conta a opinião de especialistas para assegurar que estejam em conformidade com as práticas recomendadas^(22)^, conforme demonstrado neste estudo. Além disso, o desenvolvimento desses cenários deve ser cuidadosamente planejado e sistematizado, empregando instrumentos e ferramentas que ajudem o professor/facilitador durante a simulação^(23)^.

Destaca-se ainda a importância de garantir que cada etapa do cenário esteja em concordância durante o processo de validação. Isso oferece a vantagem de criar um cenário clínico mais realista e alinhado ao tema proposto^(19)^. Neste contexto, a validação do *checklist* realizada pelos *experts* conferiu mais credibilidade ao cenário, aproximando-o ainda mais da realidade.

No decorrer da execução e filmagem do cenário para sua validação, três etapas foram colocadas em prática: *briefing*; desenvolvimento do cenário; e *debriefing*. Durante o *briefing*, primeira etapa da simulação, conforme já descrito, é importante a apresentação do ambiente, objetivos, tempo e apresentação do caso aos participantes. Essas informações devem ser claras e objetivas, aproximando o estudante/profissional à realidade simulada^(24,25)^. Já durante o cenário em ação, é essencial oferecer apoio e pista aos participantes, para que atinjam o objetivo proposto da simulação. Neste ínterim, é imprescindível atualizar os participantes sobre o progresso do caso clínico e as circunstâncias que o usuário/paciente se encontra naquele momento, para que o participante possa entender suas prioridades^(26)^.

A simulação é reconhecida como um ambiente controlado que não oferece riscos ao paciente, assemelhando-se muito à realidade. Isso tem contribuído significativamente para que os estudantes reconheçam suas limitações e compreendam a importância de adquirir um conhecimento teórico sólido essencial para fundamentar a prática^(27)^.

No *debriefing*, última etapa do cenário, cabe ao professor/facilitador sensibilizar os estudantes para compartilhar vivências e construir novos saberes^(25,28)^. Neste momento, é imprescindível identificar as emoções, retirar as dúvidas, destacar as habilidades e restrições experimentadas pelos participantes durante a situação clínica e, simultaneamente, fomentar a integração entre teoria e prática, gerando maior interesse pelo assunto discutido e reflexão sobre a estratégia aplicada no cenário clínico^(29)^. Assim, durante o *debriefing*, a discussão abordou: a comunicação e o trabalho em equipe em relação à passagem de plantão; a apresentação pessoal; a realização do exame físico; a avaliação da queixa do paciente; a análise de prontuário; a segurança do paciente em relação à higienização das mãos; a administração de medicamentos; a identificação correta do paciente; a comunicação eficaz entre paciente/familiar e equipe; a resolutividade do problema apresentado; e a evolução de enfermagem.

Ao sentir-se apoiado durante a simulação, o estudante tem mais facilidade para explorar e solucionar as questões apresentadas no cenário. O retorno do docente é de grande importância, pois permite que os mesmos reflitam sobre suas práticas e futuras atitudes profissionais^(30)^. Desse modo, a experiência vivenciada pelos estudantes durante a simulação pode conduzir a um entendimento aprofundado das possíveis circunstâncias que podem surgir no trabalho do enfermeiro, e esta experiência, realizada em ambiente controlado, próximo ao real, corrige as falhas e promove mais segurança para atuar no campo prático^(31)^.

Há uma série de vantagens ao adotar estratégias de ensino baseadas em metodologias ativas, como a simulação, que não requerem necessariamente alta tecnologia para assegurar o êxito do aprendizado^(32)^. Ainda, métodos tradicionais e simulações podem ser implementados em conjunto, auxiliando o estudante a se sentir mais confiante e seguro para atuar na prática^(33)^. Deste modo, ressalta-se a importância da divulgação de cenários clínicos validados, para que as IES possam utilizá-los nas suas respectivas disciplinas, visando ainda mais à aprendizagem do estudante^(12)^.

### Limitações do estudo

Apresenta-se como limitação um cenário específico do setor hospitalar, o que pode limitar sua aplicação em outros campos de assistência à saúde. No entanto, a comunicação e o trabalho em equipe são competências fundamentais do enfermeiro em toda a sua atuação profissional. Outra limitação se deve ao fato de o cenário não ter sido testado/validado pelos *experts* presencialmente no laboratório de habilidades/simulação junto aos discentes de graduação.

### Contribuições para a área da enfermagem

Este estudo colabora com o desenvolvimento das premissas científicas, uma vez que possibilita que os cursos de graduação em enfermagem apliquem com os discentes um cenário validado com ênfase na comunicação e no trabalho em equipe, permitindo a expansão dos seus conhecimentos em uma vivência prática. Ademais, essa situação pode oferecer uma perspectiva mais abrangente do trabalho do enfermeiro, que ultrapassa a assistência, conduzindo esses futuros profissionais a uma práxis segura e colaborativa nas instituições de saúde.

## CONCLUSÕES

Os cenários simulados se destacam como um recurso pedagógico eficaz que pode ser integrado ao currículo educacional, auxiliando o estudante na sua prática profissional. Portanto, este estudo apresentou a elaboração/criação e validação de simulação sobre as competências interprofissionais de comunicação e trabalho em equipe do enfermeiro no ambiente hospitalar. A validação, efetivada por especialistas, demonstrou a anuência das avaliações realizadas. O cenário e o *checklist* poderão ser empregados gratuitamente no ensino em enfermagem.

## Data Availability

Os dados da pesquisa estão disponíveis no corpo do artigo.
